# A feasibility study incorporating a pilot randomised controlled trial of oral feeding plus pre-treatment gastrostomy tube versus oral feeding plus as-needed nasogastric tube feeding in patients undergoing chemoradiation for head and neck cancer (TUBE trial): study protocol

**DOI:** 10.1186/s40814-016-0069-8

**Published:** 2016-06-16

**Authors:** Vinidh Paleri, Joshua Wood, Joanne Patterson, Deborah D. Stocken, Mike Cole, Luke Vale, Jeremy Franks, Teresa Guerrero-Urbano, Rachael Donnelly, Stewart Barclay, Tim Rapley, Nikki Rousseau

**Affiliations:** 1 Department of Otolaryngology–Head and Neck Surgery, Newcastle upon Tyne Hospitals NHS Foundation Trust, Newcastle upon Tyne, UK; 2 University of Manchester, Manchester, UK; 3 City Hospitals Sunderland NHS Foundation Trust, Newcastle upon Tyne, UK; 4 Clinical Trials and Biostatistics, Institute of Health and Society, Newcastle University, Newcastle upon Tyne, UK; 5 Institute of Health and Society, Newcastle University, Newcastle upon Tyne, UK; 6 Health Economics, Newcastle University, Newcastle upon Tyne, UK; 7 Guy’s & St Thomas’ NHS Foundation Trust, London, UK; 8 Restorative Dentistry, Newcastle Dental Hospital, Newcastle upon Tyne, UK

**Keywords:** Nasogastric tube, Gastrostomy, Swallow outcome, Chemoradiation therapy, Head and neck cancer

## Abstract

**Background:**

There are 7000 new cases of head and neck squamous cell cancers (HNSCC) treated by the NHS each year. Stage III and IV HNSCC can be treated non-surgically by radio therapy (RT) or chemoradiation therapy (CRT). CRT can affect eating and drinking through a range of side effects with 90 % of patients undergoing this treatment requiring nutritional support via gastrostomy (G) or nasogastric (NG) tube feeding.

Long-term dysphagia following CRT is a primary concern for patients. The effect of enteral feeding routes on swallowing function is not well understood, and the two feeding methods have, to date, not been compared to assess which leads to a better patient outcome.

The purpose of this study is to explore the feasibility of conducting a randomised controlled trial (RCT) comparing these two options with particular emphasis on patient willingness to be randomised and clinician willingness to approach eligible patients.

**Methods/design:**

This is a mixed methods multicentre study to establish the feasibility of a randomised controlled trial comparing oral feeding plus pre-treatment gastrostomy versus oral feeding plus as required nasogastric tube feeding in patients with HNSCC. A total of 60 participants will be randomised to the two arms of the study (1:1 ratio). The primary outcome of feasibility is a composite of recruitment (willingness to randomise and be randomised) and retention. A qualitative process evaluation investigating patient, family and friends and staff experiences of trial participation will also be conducted alongside an economic modelling exercise to synthesise available evidence and provide estimates of cost-effectiveness and value of information. Participants will be assessed at baseline (pre-randomisation), during CRT weekly, 3 months and 6 months.

**Discussion:**

Clinicians are in equipoise over the enteral feeding options for patients being treated with CRT. Swallowing outcomes have been identified as a top priority for patients following treatment and this trial would inform a future larger scale RCT in this area to inform best practice.

**Trial registration:**

ISRCTN48569216

## Background

Over 7000 new head and neck squamous cell cancers (HNSCC) are treated by the NHS every year. Patients with oropharyngeal HNSCC form the major group of patients who will be eligible for this research project. The incidence of oropharyngeal cancer in the UK has more than doubled in the 10 years between 1995 and 2006 [1]. In Scotland, oropharyngeal cancer is the fastest rising of all cancers [2]. In the USA, it is estimated that in 2020, oropharyngeal cancer will be more common than cancer of the uterine cervix [3].

Advanced (stages III and IV) HNSCC are now treated non-surgically by radiation therapy (RT), or chemoradiation therapy (CRT). In CRT, chemotherapy is delivered concurrently with RT, potentiating not only tumour kill but also toxicity, consequently profoundly affecting eating and drinking by causing a range of side effects: loss of taste, dry mouth, pain, loss of appetite and impaired swallow mechanism. Over 90 % of patients receiving this treatment option need nutritional support for severe dysphagia and weight loss both during and after treatment. When necessary, nutritional support can be delivered through a pre-treatment gastrostomy (G) tube or nasogastric (NG) tube feeds.

Some clinicians advocate that patients with adequate pre-treatment swallow function and oral intake have pre-treatment G tubes and continue with oral diet during treatment until they are no longer able to take adequate amounts of oral diet to maintain nutritional status. Conversely, others offer patients with adequate pre-treatment swallow function the option of continued oral feeding, until they are unable to take adequate oral nutrition to maintain nutritional status and then proceed with (reactive) passage of an NG tube as and when necessary [4, 5]. Generic guidance suggests that G tubes should be placed in patients that need enteral tube feeding for more than 4 weeks [6]. Approximately 2500 gastrostomies are performed for HNSCC patients in the UK. The insertion costs alone are approximately £3 million per annum.

G-tube placement is an invasive procedure with a small but defined risk of acute serious complications [7]; 25 to 35 % of patients retain the tube for >1 year after CRT, 10 % >2 years [8], although recent studies show reduced dependence rates [9] A G-tube has a major impact on patients’ and carers’ quality of life (QoL) [10, 11], due to leakage, soiling of clothes, and interference with family life, intimate relationships and hobbies [12]. While NG tube placement is relatively simple, the smaller diameter tube makes it prone to blockage, thus needing repeated replacement. When care is not taken to ensure correct placement, NG tube misplacement in the lungs and subsequent feeding can lead to significant morbidity, now categorised as a ‘never event’ by the Department of Health [13]. Systematic reviews fail to demonstrate evidence for functional, nutritional, quality of life or health economic benefit of either approach [14, 15]. UK practice is correspondingly variable and no robust data are available [16]. Both NG and G tube users need community support, with greater needs for NG tube users. The National Patient Safety Agency recommends that a full multidisciplinary supported risk assessment should be made and documented, before a patient with a nasogastric tube is discharged from acute care to the community. There is evidence that clinicians in some areas opt for G-tubes due to barriers to the delivery of NG tube nutritional support in the community. However, a recent British Society of Gastroenterology survey showed that only 64 % of G-tube services offer an aftercare service [17].

Long-term dysphagia is now recognised as the principal functional consequence of CRT for HNSCC, and patients report this as a top concern [15, 18]. Dysphagic patients and those dependent on tube feeds (G and NG tubes) need significant long-term supportive care and suffer from impaired quality of life [10]. The effect of either enteral feeding route on the swallowing outcome is not well understood. Gastrostomy placement reduces the need for the patient undergoing CRT to swallow to maintain nutrition. Thus, it is likely that patients using gastrostomy tubes experience a reduction in use of the swallowing musculature. This reduction combined with the mucositis caused by radiation has been hypothesised to increase the risk of fibrosis in the muscles and pharyngoesophageal stricture.

The most severe CRT reaction that causes dysphagia is complete closure of the gullet, devastating for the individual and with substantial costs for the NHS. While this risk may be higher with G-tube use, which bypasses the gullet, unlike an NG tube which maintains a degree of oesophageal patency, G-tube use is associated with shorter hospital stay and fewer hospital admissions [19]. Reconstruction requires complex major reconstructive surgery of the upper aerodigestive tract—with direct care costs of ≈£32,000 per patient [20] and a significant morbidity for the patients involved. There are national guidelines recommending that the proportion of HNSCC patients treated by intensity modulated radiation therapy (IMRT) be increased [21]. However, with respect to swallowing outcomes, IMRT has been shown to increase stricture rates 3.3 times [22]; up to 46 % of HNSCC patients treated by IMRT may needed oesophageal dilatation [23], an intervention that needs inpatient care, is distressing to the patient and is associated with complications.

A systematic review [15] has suggested that feeding route during treatment may impact on the swallow performance after CRT. Four retrospective studies [24–27] one prospective study [28] and one randomised controlled trial (RCT) (with small patient numbers) [29] have identified that swallowing difficulties are more prevalent in patients receiving a prophylactic G-tube, even in the long term. However, existing research on the association of early G-tube feeding and long-term swallow impairment has been inconclusive due to small participant numbers, by the use of insensitive dysphagia measurements and by limited long-term follow-up. The sole RCT [29] recruited from a single Australian centre in an area of low population density. A Cochrane review [14] identified no further eligible trials and concluded that there was insufficient evidence to determine the optimal method of enteral feeding for patients with HNSCC receiving RT or CRT.

These two methods have never been properly compared to establish which leads to better outcomes for patients, despite calls for better information to guide patient and clinician decisions [14, 24, 29]. We therefore wish to conduct a RCT to compare the two feeding methods (pre-treatment gastrostomy versus oral feeding plus as-needed nasogastric tube) in patients with no or only minimal swallowing problems. A similar trial in Australia [29] failed to recruit enough patients; hence, we wish to first carry out a feasibility study to see whether a RCT is possible and inform how it should be conducted.

## Methods/design

### Aims and objectives

Our aim is to determine whether a definitive RCT is feasible in head and neck cancer patients with minimal swallowing problems undergoing CRT comparing oral feeding plus prophylactic gastrostomy tube feeding versus oral feeding plus as-needed nasogastric tube feeding (TUBE study). The TUBE study feasibility phase is a necessary prelude to a full trial of these complex interventions, to assess (i) whether an adequate proportion of eligible patients can be recruited into the study and (ii) whether an adequate proportion of patients comply with the trial protocol including outcome measure completion, according to both quantitative and qualitative data.

The objectives are:

A. To explore barriers and facilitators to trial implementation and to use this information to improve recruitment and retention
  1. Willingness of participants to be randomised, to accept and persist with allocated treatment and comply with assessments.
  2. Willingness of clinicians (including clinical oncologists, surgeons, nutritionists, speech and language therapists) to recruit patients.
  3. Qualitative assessment of patient and carer perspectives on trial participation, barriers to randomisation among non-participants, acceptability of assessment tools and experience of the tube feeding, conduct and compliance of the trial protocol and reasons (and characteristics) of patients dropping out.
B. To carry out preliminary estimation of key parameters to inform definitive study design and study processes
  1. Confirm primary outcome measure and associated power/sample size for definitive trial: dysphagia related quality of life as measured by MD Anderson Dysphagia Inventory (MDADI) HNSCC-specific self report scale (variation and differences in change from baseline at 6 months).
  2. Trial our subsidiary QoL outcomes (the European Organization for Research and Treatment of Cancer questionnaires EORTC QLQ-C30, EORTC QLQ - H&N35), short form 36 (SF-36) a multi-purpose, short-form health survey and data collection tools for use of health and personal social services and patient costs.
  3. Monitor nutritional parameters: body mass index, weekly weight changes (during treatment) and quantity of enteral nutrition consumed.
  4. Derivation of an algorithm for when NG tube should be placed in the oral intake arm that is acceptable to patients and nutritionists.

C. Provide health economics metrics
  1. Assess economic value of information based upon a modelling exercise informed by the feasibility study and the existing systematic reviews.
  2. Provide preliminary estimate of the costs, effects and relative cost-effectiveness of the alternative methods of nutritional support based upon the modelling exercise.

### Design

A mixed methods multicentre study to establish the feasibility of a RCT of feeding methods in patients with stages III and IV head and neck cancer receiving CRT with curative intent. The work will be conducted over 24 months.

The components are:

1. A multicentre randomised controlled pilot feasibility trial comparing oral feeding plus pre-treatment gastrostomy versus oral feeding plus as required nasogastric tube feeding in patients with HNSCC. Patients will be randomised on a 1:1 basis and stratified by IMRT and by the treating centre.
2. A qualitative process evaluation to inform the trial design by investigating patient, carer and staff experiences of trial participation.
3. A modelling exercise to synthesise available evidence and provide preliminary estimates of cost-effectiveness and value of information from future research.

### Setting

The setting was five tertiary NHS centres for HNSCC. For our definitive trial, additional centres will be recruited via our links with the National Cancer Research Network, Comprehensive Clinical Research Network and from respondents to our national survey [16].

#### Ethical considerations

Ethical approval was sought and granted by the Newcastle and North Tyneside 2 Committee, NHS Health Research Authority, reference 14/NE/0045.

### Target population

The target population will be patients with stage III and IV HNSCC who receive primary CRT with curative intent.

Inclusion criteria:

(1) Grade 1 pre-treatment dysphagia, as defined by Common Terminology Criteria for Adverse Events v4.0 (defined as asymptomatic/symptomatic/able to eat regular diet). (2) Consent to be randomised.

Exclusion criteria:

Patients who (1) decline to participate, (2) are unable to give informed consent, (3) cannot receive a gastrostomy for medical reasons, (4) do not receive treatment with curative intent and (5) patients with malnutrition requiring immediate initiation of enteral feeding.

Clinical experience suggests that patients with primary sites in the oropharynx, larynx, nasopharynx and unknown primaries are those who will fulfil the inclusion criteria (~35 to 40 % of all HNSCC patients).

### Interventions

Pre-CRT gastrostomy arm: endoscopic or radiologic gastrostomy will be placed before commencement of CRT. Given the pragmatic nature of this study and equivalent success rates with either technique, the choice of method of insertion will be left to local protocols of the treating centre. Patients will continue with oral feeding and commence use liquid nutrition through the G tube when they are unable to maintain an adequate oral intake to meet their nutritional requirements (<75 % of requirements based on a dietetic assessment of 24-h recall by patients).

No pre-CRT gastrostomy arm: this group will continue with oral feeding or have a nasogastric tube if required during treatment. The decision to place a nasogastric tube will be based on clinical assessment, patient request and published guidelines [6], patient request for gastrostomy tube may also lead to gastrostomy placement. Based on published prospective data, we anticipate that <10 % will convert from NG tube to G tube during the study.

### Outcomes measures

The primary outcome of feasibility will comprise of the following entities:

1. Willingness of patients to be randomised (assessed via review of patient screening logs) defined as:
  i. The number of patients consenting to be randomised as a proportion of all patients approached about the trial, with reasons for non-consent
  ii. Qualitative assessment of barriers and facilitators to recruitment
2. Willingness of clinicians to randomise patients—assessed via qualitative interviews
3. Assessment of retention and drop-out rates, defined as:
  i. The number of patients who start randomised treatment as a proportion of the number randomised, with reasons for early drop outs
  ii. The number of patients who complete randomised treatment as a proportion of the number randomised, with reasons for drop outs (including death)
  iii. Completeness of MD Anderson Dysphagia Inventory (MDADI) assessment within 6 months
  iv. Qualitative assessment of barriers and facilitators to data collection and participant retention

Secondary outcome measures are:

The following scores/ measures will be requested at baseline (before treatment) and at 3 and 6 months after treatment (5 and 8 months from randomisation):

A. The MDADI is a self-administered questionnaire designed specifically for evaluating the impact of dysphagia on the quality of life of patients with head and neck cancers [30]. This will be our primary outcome measure in the full trial.
B. Quality-of-life outcomes

We will use two questionnaires to assess quality of life outcomes. The European Organization for Research and Treatment of Cancer questionnaire EORTC QLQ-C30 (version 3.0) is a cancer-specific instrument, has a multidimensional structure, appropriate for self-administration and is applicable across a range of cultural settings [31]. The EORTC QLQ - H&N35 is the head and neck module for EORTC QLQ-C30 (version 3.0) and is intended for use in conjunction with the QLQ-C30 in patients with head and neck cancer [32].

C. Short form 36

The SF-36 is a multi-purpose, short-form health survey with 36 questions. It yields an eight-scale profile of functional health and well-being scores as well as psychometrically based physical and mental health summary measures and a preference-based health utility index that will be used to derive quality-adjusted life-years for a cost-utility analysis conducted as part of a subsequent full trial.

D. Use of health and personal social services and costs to patients and their families/carers

For the definitive economic evaluation costs to patients and their families/carers, the NHS and personal social services will be elicited. Within the feasibility study, we will develop the tools necessary to elicit these costs. Specifically, we will develop case report forms and questionnaires to capture use of services and patient/family/carer costs. The content of these forms will be developed in consultation with the study team, our existing item bank of questions, web based resources e.g. www.dirum.org and experience from other RCTs of nutritional interventions e.g. the recent SIGNET trial [33]. The tool developed will be administered at 3 and 6 months after treatment.

E. Nutritional parameters

We will measure the body mass index before treatment, at 3 months and at 6 months following treatment. We will also measure weekly weight changes during treatment and the quantity of enteral nutrition consumed.

F. Oral health monitoring

We will perform oral health assessment before commencing treatment and the end of 6 months for all dentate patients. A full dental chart with panaromic radiographs will be performed at pre-treatment and at 6 months. Periodontal and oral hygiene assessment and plaque scores will also form part of this assessment and be documented.

### Statistical considerations

Target recruitment is 60 patients (30 per arm of the study) [34]. As a feasibility study, we will provide an assessment of recruitment and compliance rates to inform future trial design. Statistical analyses are according to a confidence interval rather than a hypothesis testing approach. Analyses will be descriptive reporting rates as proportions with confidence intervals and graphical analyses of longitudinal data. Recruitment is dependent on the number of patients approached but should be no lower than 50 %. The upper limit of the 95 % confidence interval for the proportion of patients recruited should exceed 50 %. If 120 patients were approached and 50 % were recruited (60 patients randomised), the 95 % confidence interval for the recruitment rate would have width ±9 %. This would provide a good level of accuracy to assess the acceptability of the recruitment rate.

### Progression criteria to phase III trial

The decision to move to a phase III trial will be based on:

1. Adequate timely recruitment with a 50 % recruitment rate.
2. Completeness of outcome measurement (MDADI at 6 months): excluding those individuals who die during the study period. This outcome data successfully collected should be greater than or equal to 80 %, as this will be the primary outcome for the planned phase III trial.
3. Economic criteria of EVOI to suggest further research likely to be worthwhile.

#### Qualitative process evaluation

The integrated qualitative process evaluation will explore barriers and facilitators to trial implementation. Through regular feedback of emerging findings to the trial team it will inform the day-to-day running of the pilot trial and optimise recruitment and retention. It will also identify aspects of trial processes which could be improved for the definitive trial.

A. Normalisation process theory

Our analysis of facilitators and barriers to trial participation will be informed by normalisation process theory [35]. Normalisation process theory considers factors that affect implementation in relation to four key areas; how people make sense of a new practice (in this case—the trial) (coherence); the willingness of people to sign-up and commit to the new practice (cognitive participation); their ability to take on the work required of the practice (collective action); and activity undertaken to monitor and review the practice (reflexive monitoring). This theory is increasingly being used in studies of the implementation of interventions in health care (www.normalizationprocess.org). In the current study, we will consider how well trial processes are introduced and incorporated at each site for both patient and professional groups.

B. Methods and sampling

Numbers have been included to give an indication of the amount of data to be collected. We have given particular thought, for example, to whether there might be significant subgroups of patients or professionals, which would require an additional layer of data collection and analysis. However, in keeping with the principles of rigorous qualitative research, the actual sample size will be informed by the point of data saturation; we will be responsive to the study context and anticipate that in some cases, fewer interviews or observations will be conducted, and in others, additional data will be collected in response to our emerging analysis or study events. We aim to achieve a balance between spread of data (to avoid missing key events or issues) and depth (a manageable data set that allows for in-depth analysis); having the flexibility to identify and focus in on those issues which our analysis suggests is key to successful study implementation.

##### Data collection will focus on three inter-related phases over the duration of the trial

Following appropriate consent, we will include interviews with health professionals (*n* = 15–24); patients (*n* = 32–36); observation of recruitment discussions (*n* = 9–18); and observations of multidisciplinary team meetings (*n* = 6–15); details below.

##### Pre-implementation of TUBE pilot trial

In order to understand the context, professionals’ views and expectations and to map patient pathways, we will visit each study site. We will conduct formal interviews with key individuals (*n* = 2–3 per site) involved in treatment planning for HNSCC (e.g. nurses; speech therapists; ENT surgeons).

##### Patient recruitment phase

We will focus on patients’ and professionals’ experiences of recruitment. Patients will be recruited from multidisciplinary clinics, with trial information being provided by research nurses. We will conduct qualitative interviews with patients who have consented to take part in the trial (*n* = 5 in the G-tube group; *n* = 5 in oral feeding group) and where possible, those that decline to take part either at recruitment or who withdraw at the point of randomisation (*n* = 10). We will focus on their experiences and understandings of trial processes (e.g. the information they were given; the recruitment encounter; their ideas and/or concerns about randomisation and consent) and the intervention (willingness to undergo either feeding option; ideas and/or concerns about impact on health and acceptability).

Ideally, we would interview patients within 2 weeks of recruitment discussions. Patients will be offered a choice of location and method (telephone; face-to-face) of interview. We know that individuals often discuss clinical decisions with other people [36] so patients who wish to involve a family member in their interview will be able to do so.

We will also conduct further interviews with professionals involved in some aspect of patient recruitment (*n* = 3–5 per site) and, where possible, observe multidisciplinary team meetings to understand the process of identification of eligible patients (*n* = 1–3 per site). Again, where possible, study recruitment discussions (either at treatment planning clinic or subsequent discussion with clinical nurse specialist) will, with consent, be observed (*n* = 3–6 per site). An initial visit to each site will take place shortly after (1–2 months) the site commences patient recruitment. The timing of subsequent visits to a site will be driven by factors such as variation in recruitment rates between sites or changes in key personnel.

#### Patient follow-up phase

We will also investigate patients’ (and carers’) experiences of the tube feeding and trial participation through qualitative interviews (*n* = 12–16) at approximately 8 months after recruitment, in order to explore the acceptability of assessment tools and their experiences of the intervention. Where possible, the follow-up interviews will include second interviews with patients and family members (or friends) interviewed at the recruitment phase; additional participants will be recruited based on emergent criteria (e.g. length of time of oral and/or supplementary feeding).

C. Qualitative data management and analysis

Interviews and recruitment discussions will, with consent, be audio-recorded, transcribed verbatim and edited to ensure anonymity of respondent. Data will be managed using NVivo software. The analysis will be theoretically-informed by normalisation process theory and will be conducted according to the standard procedures of rigorous qualitative analysis [37] including open and focused coding, constant comparison, memoing [38], deviant case analysis [39] and mapping [40]. We will undertake independent coding and cross checking and a proportion of data will be analysed collectively in ‘data clinics’ where the research team share and exchange interpretations of key issues emerging from the data.

D. Relationship between process evaluation and pilot trial

Findings will be regularly fed back to the study team and appropriate changes made to study processes during the lifetime of the study. For example, if the pre-pilot work identifies a problem with the coherence of the trial to one group of professionals, then additional awareness raising/education sessions or materials will be developed.

#### Economic modelling

The economic model will estimate the costs, effects and relative cost-effectiveness of the alternative methods of nutritional support. Data collection takes place during the study and all analyses will be conducted by health economists at the end of the study period. It will take the perspective of the UK NHS and personal social services (PSS). In the base case, all costs and effects will be discounted at 3.5 % [41]. In addition, as described below, the model will also estimate the expected value of information and expected value of sampling information to help inform the future definitive study.

The methods to paramaterise the model are described below. To allow for uncertainty to be characterised around these data—costs, effects and cost-effectiveness—and to enable the value of information analysis to be conducted a probabilistic sensitivity analysis will be performed using Monte Carlo simulation. All uncertainty surrounding estimates of input parameters will be informed by appropriate distributions. Modelling will conform to recommendations for best practice including those developed for economic evaluation models [42].

A. Structure of the economic model

Treatment pathways for patients undergoing chemoradiation for HNSCC will be developed. The treatment pathway will start with the choice of nutritional support. These pathways will be based upon the material prepared for the feasibility study and advice from the key stakeholders involved in the study. Following recommendations for best practice, these care pathways will be used to develop a mathematical model covering the period of initial intervention and the costs and consequences of any subsequent outcomes including further interventions.

B. Derivation of cost data

Information on the precise description of the resources required for each intervention will be based upon data derived from the feasibility study. From participants recruited to the trial, we will estimate costs for each method of nutritional support. Given the small study size, these data will be imprecise and this imprecision will form a key input into the analysis. Further cost data will be required on subsequent management; these data will be identified with the help of members of the study group and a search of the economic literature. Unit costs will be taken from appropriate routine sources e.g. NHS reference costs, British National Formulary for drugs.

C. Derivation of utilities

For the cost-utility analysis, effects/benefits will be estimated in quality-adjusted life-years. For each health state, a health state utility will be defined. The data will be derived from the pilot trial and a focused search to identify utility data, including a search on the CEA Registry (https://research.tufts-nemc.org/cear/default.aspx). The estimates used within the model will be based upon the best available data, ideally derived using SF-6D (as the SF-36 will be used within the feasibility study and can be used to derive SF-6D scores). Nevertheless, such data may not be readily identifiable and data from other sources along with judgements as to how that measure compares to SF-6D scores will be used.

D. Epidemiological and relative effectiveness data

The main source of evidence to inform the probabilities required for the model will be the existing systematic reviews and other literature summarised in the ‘Background’ section of this application. From these sources, information on the likelihood of key events described in the economic model will be sought. Additional focused searches will be conducted as necessary to identify the best available evidence relevant to the UK NHS for such probabilities.

E. Estimation of relative efficiency

The results of the economic model will be presented as a cost-utility analysis (CUA). In the CUA, mean costs, mean quality-adjusted life-years, incremental costs and quality-adjusted life-years will be presented.

F. Uncertainty

It is possible that sufficient data to populate the model will not be identified. In such a case, threshold values will be explored where data is missing by varying estimates through a range thought plausible (based on advice of the stakeholders involved in the study). Within a probabilistic analysis, a plausible distribution will be assigned to this range (which may include a uniform distribution to indicate that we do not know what value a parameter might take within a specified range).

Deterministic sensitivity analyses will be carried out to test for the effect of assumptions and variability [43] such as the impact of changes in discount rates. The probabilistic sensitivity analysis will also be undertaken for both the base case analysis and, where sensible, all deterministic sensitivity analyses allowing presentation of results in a series of cost-effectiveness acceptability curves (CEAC). Estimates of costs and quality-adjusted life-years will be calculated as the expectation over the joint distribution of the parameters. Relevant distributions will be informed by the systematic reviews and meta-analyses, other literature or expert opinion according to best practice [44].

G. Value of information analysis: Identification of future research needs

We anticipate that the economic model will only provide preliminary data on the relative effectiveness and cost-effectiveness of the different methods of nutritional support. The main purpose of the model will be to help inform decisions about the direction of future research. Within the economic component of this study this will be explored using variants of value of information analysis [45–48].

We will initially estimate the expected value of perfect information and expected value of removing uncertainty surrounding specific parameters or groups of parameters to identify where further research should focus on identifying more precise and reliable estimates of specific pieces of information e.g. relative effectiveness, costs, utilities (the expected value of partial perfect information). Expected value of perfect information and expected value of partial perfect information can be interpreted as the value of eliminating a wrong decision and it places an upper value on conducting further research overall (expected value of perfect information) or a specific area of information (expected value of partial perfect information). Expected value of perfect information and expected value of partial perfect information at an individual level can be estimated directly from the model but will need to be combined with information on the number of people who could benefit from the gastrostomy tube feeding over the expected lifetime of the project. As these two factors are uncertain sensitivity analysis will be used to explore alternative assumptions. If relatively small values are obtained for expected value of perfect information and expected value of partial perfect information (although we note that this is a judgement), then this suggests that no further research is necessary or no further research is required to obtain ‘better’ estimates for specific groups of parameters.

A judgement will be formed based upon the findings the expected value of perfect information analysis as to whether a move to the analytically complex, expected value of sampling information, will be made. Expected value of sampling information provides further information on the value of removing some of the existing uncertainty and also explicitly takes into account the cost of generating that future research to estimate the expected net benefit of sampling. Specifically, in this study, we will explore the use of this approach to identify, from an economic perspective, the optimal trial design. Such methods have only very rarely been used to determine the size of randomised controlled trials and therefore following the recommendations of forthcoming guidance the findings of this analysis will be used along with other information to determine the sample size for the trial [49].

## Discussion

### Potential impact of the trial

Our national survey [16] shows that clinicians working in this field are in equipoise regarding the feeding route to be used in patients receiving chemoradiation for head and neck cancer. The survey concluded that in the absence of a national consensus on which patients to recommend for gastrostomy, consideration should be given to the development of clinical decision-making models in an attempt to systematise the decision-making process.

Clinicians have looked at this issue in a paper published in 2001 [24], when a robust study was called for in this field. Since then, Cochrane reviews [14] and other prospective and retrospective studies [25, 27–29] have been published, indicating that there is an appetite to progress the science in this area. By using swallowing outcomes as our primary measure, we are focusing on an outcome that patients tell us as being important issue [18]. Thus, if this trial finds evidence to support a change in clinical practice, such findings will have the required impact. It is worth noting that if the change in practice were to support the non-surgical (oral + NG tube) arm, there may be reductions in morbidity and mortality caused by the interventions. There may also be potential cost savings in the non-surgical arm but this may be offset by the cost of repeat return to hospital for re-siting NG tubes, radiologic examinations, and the cost of district nursing support with NG tubes which is not always required with G tubes. In addition, some of the generic conclusions on the patient and carer experiences of tube feeding will be applicable to other areas of medicine. Thus, research on this area may serve to direct resources appropriately, reduce unnecessary interventions and thus reduce morbidity, mortality and improving swallowing outcomes. Irrespective of the effect size the TUBE study demonstrates, the project will generate a great deal of invaluable comparator information about the two treatments which can inform patient contribution to future shared decision-making. Our integrated qualitative study will draw on the theoretical framework of normalisation process theory [35] to identify the barriers and facilitators to changing clinical practice in this field.

### Trial status

Currently recruiting/in-patient follow-up.

## Abbreviations

CEA, cost-effectiveness analysis; CEAC, cost-effectiveness acceptability curves; CRT, chemoradiation therapy; CUA, cost-utility analysis; EORTC, European Organization for Research and Treatment of Cancer; G, gastrostomy; HNSCC, head and neck squamous cell cancer; IMRT, intensity modulated radiotherapy; MDADI, MD Anderson Dysphagia Inventory; NG, nasogastric; PSS, personal social service; QoL, quality of life; RCT, randomised controlled trial; RT, radiation therapy; SF-36, short form 36.
